# Medical Service during War and Peace

**Published:** 1915-10-30

**Authors:** 


### MEDICAL SERVICE DURING WAR AND PEACE.

In a recent address Sir William Osler, in his usual graphic manner, discussed the relations between war and science, and while recognising that the terribly destructive character of modern warfare is the outcome of scientific investigations and inventions, he showed, not less clearly, that the efficient care of the sick and wounded owes its origin to the same influence. It is certain that Science can command no direct influence which will make wars to cease, and that all her contributions to the mechanism of slaughter, however offensive in form, are readily presented as defensive in substance. There remains only the attempt to multiply the means by which the sufferings inflicted by scientific skill can be, at least in some measure, by scientific skill redeemed. Such a conclusion means in practice the organisation of preventive and curative medicine to military ends. It obviously has at its basis a humane motive which makes a wide appeal to one of the elementary sympathies of human nature.

What is specially worthy of note is the enormous difference, in the relations of the Medical Service, between the claims created by war and those existing in times of peace. Such difference, of course, exists also in reference to the combatant forces, but it is especially conspicuous in the case of the medical officers and organisations. Drilling and manœuvring and schemes of attack and defence can be applied in peace time with some approach to the conditions attending a state of war, but it is in war alone that the Army doctors have to face the claims of thousands of casualties and have to conduct operative and other procedures subject to the predominating claim of military necessity. From this it follows that in so far as preparation is possible the Army doctors have to make it in circumstances which are far removed from the field of reality, and this has been so far realised that of recent years these officers have been expected at regular intervals to attend special courses of study arranged for them in civil hospitals. In other words, their opportunities in times of peace are particularly limited in relation to the claims which fall upon them when war conditions prevail. A second consequence of the wide gap which separates war from peace in the life of the medical department of an army is the need for prompt expansion when war begins. This has been forcibly illustrated by recent experience in this country, where civil hospitals, doctors, and nurses have had rapidly to transform themselves to meet the suddenly increased demands of the Army.

If as a general truth the nation must support its fighting soldiers, so in a very special sense must the civil organisation of the profession be linked to the Army doctors, and as the Army gradually extends to become the nation in arms, so must the resources of the whole profession become available directly or indirectly for national purposes. To meet this position there is necessarily official machinery, and it is quite right that this machinery should be expected to make provision for the supply of what is necessary to render the medical services of the Army efficient in all its various branches. Yet, whatever judgment may be passed on voluntary efforts in other directions, it is certain that here the public believes emphatically in the advantages of schemes and efforts outside the official proposals. This faith expresses itself in works, which, for the time being, have come to be spoken of as Red Cross activities. The practical advantages which have been conferred by these activities on the sick and wounded in the present war can hardly be exaggerated, and they are generously and fully recognised by the military authorities. So far from being regarded with jealousy or official indifference they have been welcomed most warmly and gratefully, and indeed the co-operation of the non-official with the official organisation may be described as complete in every respect. This is not to say that the outside schemes have in every respect official sanction, or that they are invariably characterised by wise judgment or good taste. But they are fulfilling a purpose which is dear to the public heart and conscience, and no one in these circumstances will be allowed to hinder the general will by discussions on the exact manner or fashion in which the thing is being done. Husbands and sons and brothers and lovers are suffering for the country's welfare, and the people generally will not be content unless they also can do something to lessen the pain and cheer the spirit of the men who have fought for the common cause.

For the attitude of mind and sentiment which this Red Cross work expresses we are largely indebted to the existence and life of our voluntary hospital system. The natural sturdy independence of our people is doubtless at the bottom of it, but this has very largely been trained and directed by the hospital methods of the country. Not only the more comfortable members of the community who have been interested in the hospitals as philanthropic institutions, but also the poorer classes, have learned the value to the patient of individual sympathy and interest, and have come to recognise the claim of personal service, were it but the gift of a flower, in the cause of the sick and suffering. This training has for years been an increasing influence, and it is now bearing happy consequences and helping to redeem human nature from pessimism and despair. Whatever the darkness of the hour, there are still many who continue to carry the lamp.

